# Vertical Scar Mastopexy With a Centrally Based Auto-Augmentation Flap

**DOI:** 10.1093/asjof/ojac062

**Published:** 2022-07-16

**Authors:** Ryan E Austin, Morgan Yuan, Frank Lista, Pierre Lapaine, Jamil Ahmad

**Affiliations:** Division of Plastic, Reconstructive & Aesthetic Surgery, Department of Surgery, University of Toronto, Toronto, ON, Canada

## Abstract

The authors describe their technique for vertical scar mastopexy with a centrally based auto-augmentation flap. Since 2011, the authors have performed this procedure in 212 patients and found that this operative technique has allowed us to achieve reproducible aesthetic outcomes while minimizing complications. Vertical scar mastopexy with a centrally based auto-augmentation flap is an excellent procedure for patients with breast ptosis who desire improved breast shape and superior pole fullness without the use of a breast implant. The perioperative management and detailed steps of the procedure are outlined.

**Level of Evidence: 5:**

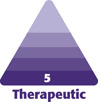

We have had significant experience with vertical scar breast reduction in our practice,^1-5^ so the adoption of a vertical scar mastopexy technique has come naturally to us. Ribeiro first introduced the concept of an auto-augmentation flap in breast reduction to help reshape the breast, using an inferiorly based auto-augmentation flap combined with an inverted-T scar pattern.^6,7^ Graf and Biggs popularized the use of an auto-augmentation flap with a vertical scar pattern, using a sling of the *pectoralis major* muscle to support the repositioned auto-augmentation flap.^8-11^ Over the past 15 years, our approach to vertical scar mastopexy with a centrally based auto-augmentation flap has evolved into the procedure we use today. Specifically:

- Repositioning of the nipple-areola complex (NAC) on a superior or superomedial dermoglandular pedicle, depending on its position with respect to the skin markings;
- Suture suspension of the centrally based auto-augmentation flap to the pectoral fascia in the superior pole of the breast using a slowly absorbing suture.

In this article, we present a detailed description of our current technique and review our experience over the past decade. The guiding principles outlined in the Declaration of Helsinki were strictly adhered to throughout the study. Written consent was provided, by which the patients agreed to the use and analysis of their data.

## PREOPERATIVE CONSIDERATIONS

Careful patient selection is critical to obtaining good aesthetic results. Preoperative education of the patient to ensure that appropriate expectations are set helps to establish the foundation for postoperative patient satisfaction. It is important for patients to understand that their anatomy and tissue quality significantly influence the aesthetic outcome. The ability to move the breast footprint superiorly is limited, and there is a limit of how much superior pole fullness can be created during mastopexy.^12,13^

The ideal candidate is a patient with adequate or excess breast volume and mild to moderate skin excess. It is also possible to excise breast tissue for patients who desire volume reduction. Patients with minimal horizontal laxity and a tight inferior pole are not good candidates, as this will tend to compound the deformity. These patients are more appropriately treated with periareolar scar mastopexy. Patients with severe skin excess may be better suited for a technique that allows for greater skin excision. In these cases, we modify the skin excision pattern to result in a J-scar to allow excision of more skin laterally.

Patients must be nonsmokers or must have quit smoking for 4 weeks before surgery. Patients on oral contraceptive pills or hormone replacement therapy are asked to discontinue use for 1 month before and after surgery to reduce the risk of venous thromboembolism (VTE). Before surgery, patients should have a stable weight with a BMI of less than 35 kg/m^2^.

In the preoperative area, patients are started on our perioperative warming protocol, which is continued intraoperatively and postoperatively.^14,15^ One hour before surgery, patients are premedicated with oral acetaminophen 1000 mg (Johnson & Johnson, Markham, ON, Canada), celecoxib 200 mg (Pfizer, Kirkland, QC, Canada), pregabalin 50 mg (Pfizer, Kirkland, QC, Canada), and ondansetron 8 mg (Novartis Pharmaceuticals Canada Inc., Dorval, QC, Canada) to minimize opioid requirements and reduce postoperative nausea and vomiting. Compression stockings and sequential compression devices are placed on the lower extremities before the induction of anesthesia to reduce the risk of VTE. Chemoprophylaxis for VTE is utilized in very high-risk patients (Caprini/Davison risk assessment model score >7) or when mastopexy is performed in combination with abdominoplasty. When indicated, chemoprophylaxis with dalteparin sodium 5000 IU (Pfizer, Kirkland, QC, Canada) is initiated on the morning of postoperative day 1 and is continued for a total duration of 14 days.

## SURGICAL TECHNIQUE

The operative sequence for vertical scar mastopexy with a centrally based auto-augmentation flap is outlined in Table 1. A detailed video demonstrating the procedure can be accessed in the Video.

**Table 1.** Operative Sequence for Vertical Scar Mastopexy With a Centrally Based Auto-Augmentation Flap

Step	Details
1	Markings
2	Infiltration
3	Liposuction (if indicated)
4	Pedicle selection to reposition the nipple-areola complex
5	Dissection of dermoglandular pedicle with a thickness of 2.5 cm
6	Dissection of centrally based auto-augmentation flap with preservation of medial and lateral parenchymal pillars
7	Dissection of subglandular pocket
8	Inferior mobilization of auto-augmentation flap
9	Adjustment for volume asymmetry by resection from auto-augmentation flap (if indicated)
10	Inset of auto-augmentation flap to pectoral fascia in subglandular pocket
11	Four-point box sutures to gather vertical wound
12	Correction of horizontal skin pleating with inverted dermal sutures
13	Inset of nipple-areola complex with inverted deep dermal and intradermal sutures
14	Skin staples to close vertical wound
15	Application of dressings and surgical bra

**Figure 1.**
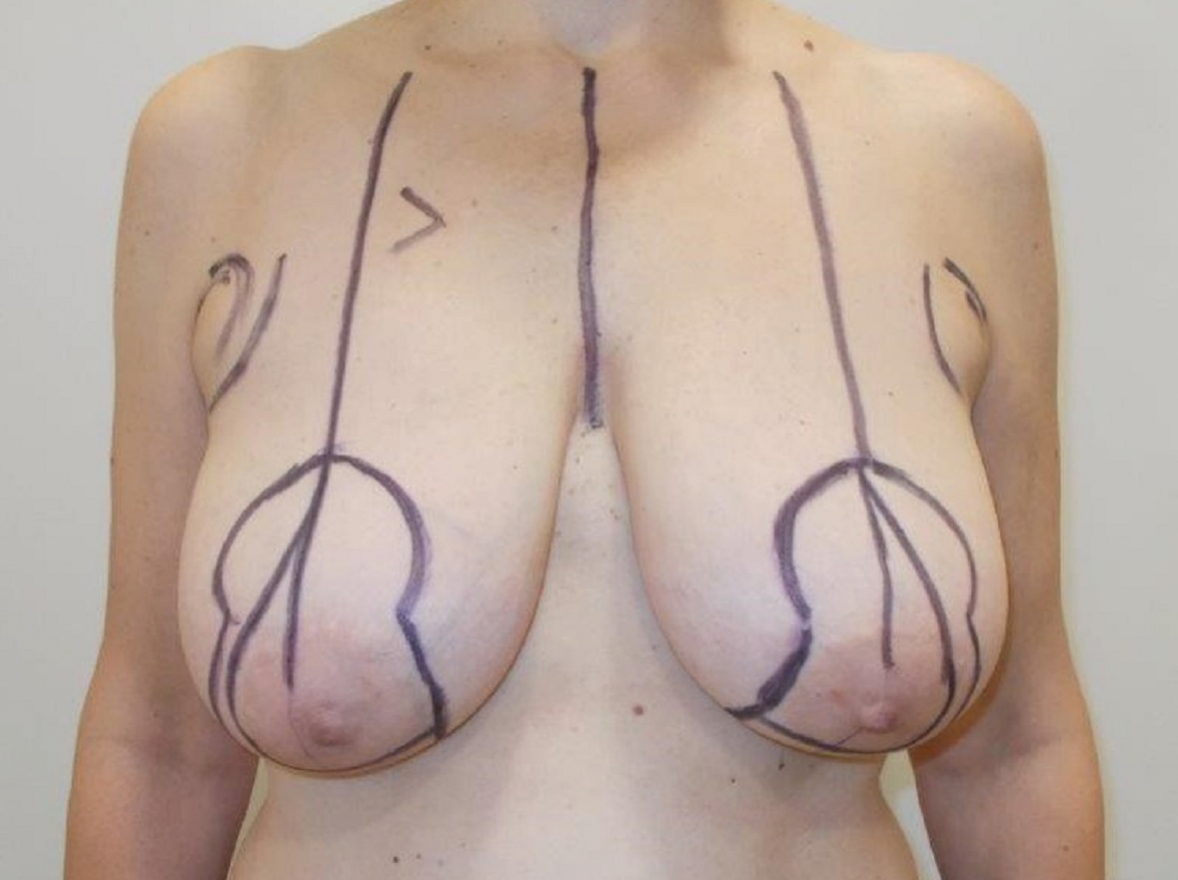
Preoperative markings of a 50-year-old female who underwent vertical scar mastopexy with a centrally based auto-augmentation flap using bilateral superomedial pedicles to reposition the nipple-areola complexes.

**Figure 2.**
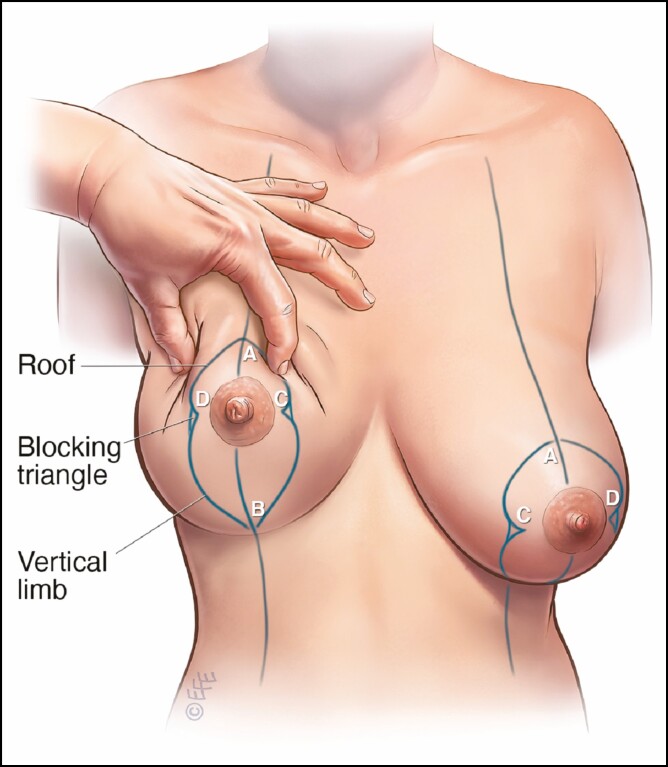
Mosque dome marking pattern: (A) new location of the superior border of the new nipple-areola complex; (B) inferior limit of the skin excision; (C, D) blocking triangles.

**Figure 3.**
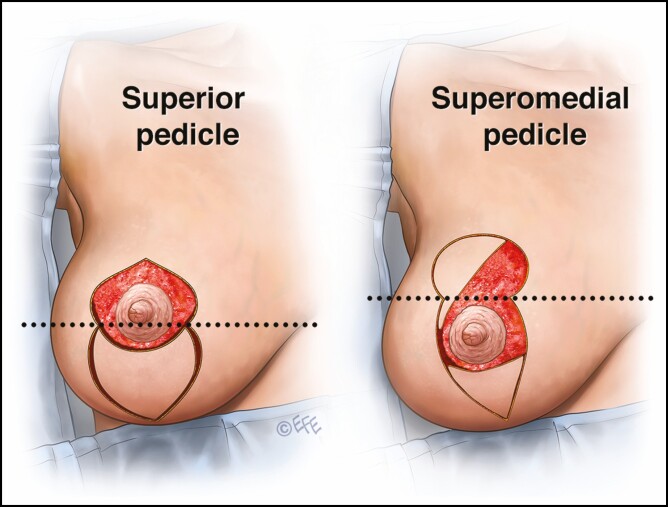
Pedicle selection. If any of the new nipple-areola complex (NAC) is located above a line joining points C and D, then a superior pedicle is designed. If the entire new NAC is located below this line, a superomedial pedicle is used.

**Figure 4.**
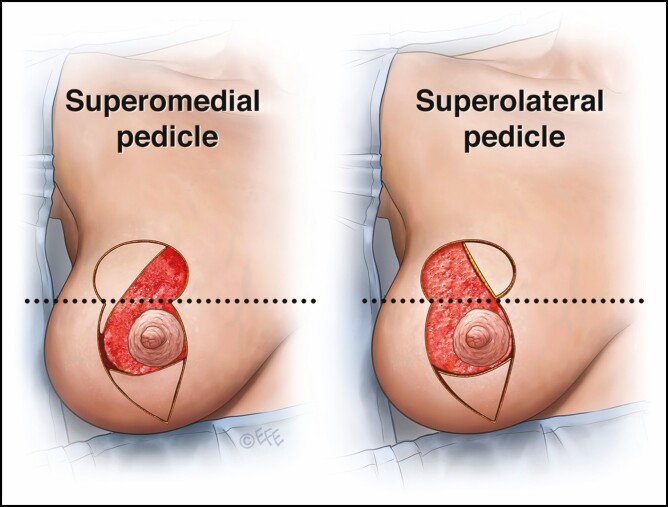
In cases where the new nipple-areola complex (NAC) is abutting the medial pillar, it cannot be transposed into the roof of the mosque dome pattern using a superomedial pedicle so a superolateral pedicle is used to facilitate the inset of the new NAC.

A mosque dome skin marking pattern is used (Figures 1, 2). A superior or superomedial pedicle is selected as previously described for our vertical scar reduction mammaplasty (Figure 3).^1-5^ In cases where the new NAC is abutting the medial pillar, it cannot be transposed into the roof of the mosque dome pattern using a superomedial pedicle so a superolateral pedicle is used to facilitate the inset of the new NAC (Figure 4).

## POSTOPERATIVE CARE

Patients continue on a multimodal postoperative oral analgesia protocol for 5 days consisting of acetaminophen 1000 mg every 8 hours, celecoxib 200 mg once daily, and pregabalin 25 mg twice daily. Hydromorphone 1-2 mg every 6 hours is used as needed. Patients receive ondansetron 8 mg 3 times daily for 1 day to reduce postoperative nausea and vomiting. Patients also take 5 pellets of Arnica montana 12C (Boiron, Saint-Bruno-de-Montarville, QC, Canada) 3 times daily for 10 days to minimize swelling and bruising.

All patients are seen for routine postoperative follow-up on postoperative day 1 and then on day 5 or 6 for removal of skin staples. They are seen at 2 weeks and 1 month postoperatively. Patients are instructed to wear their postsurgical bra for 1 month postoperatively. Starting after the removal of staples, patients begin daily breast massage exercises with arnica gel. Patients are instructed to be gentle with massage in the superior pole of the breast where the auto-augmentation flap has been inset. Scar care with silicone sheeting is initiated once the incision is completely healed. After 1 month, patients return for routine checks at 3, 6, and 12 months postoperatively.

## EXPERIENCE AND OUTCOMES

**Figure 5.**
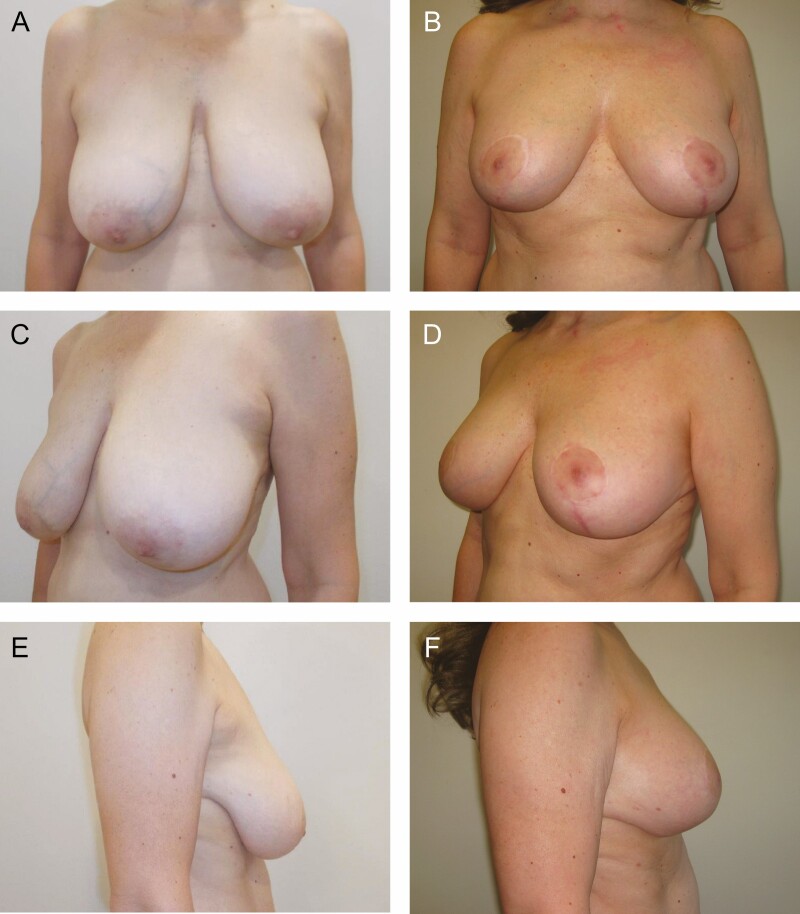
Preoperative (A, C, E) and 17-month postoperative (B, D, F) photographs of a 50-year-old female who underwent vertical scar mastopexy with a centrally based auto-augmentation flap using bilateral superomedial pedicles to reposition the nipple-areola complexes. She had 110 g excised from the right and 90 g excised from the left and liposuction of the lateral chest with aspiration of 50 mL from each side. Her preoperative markings are shown in Figure 1, and her surgery is shown in the Video.

**Figure 6.**
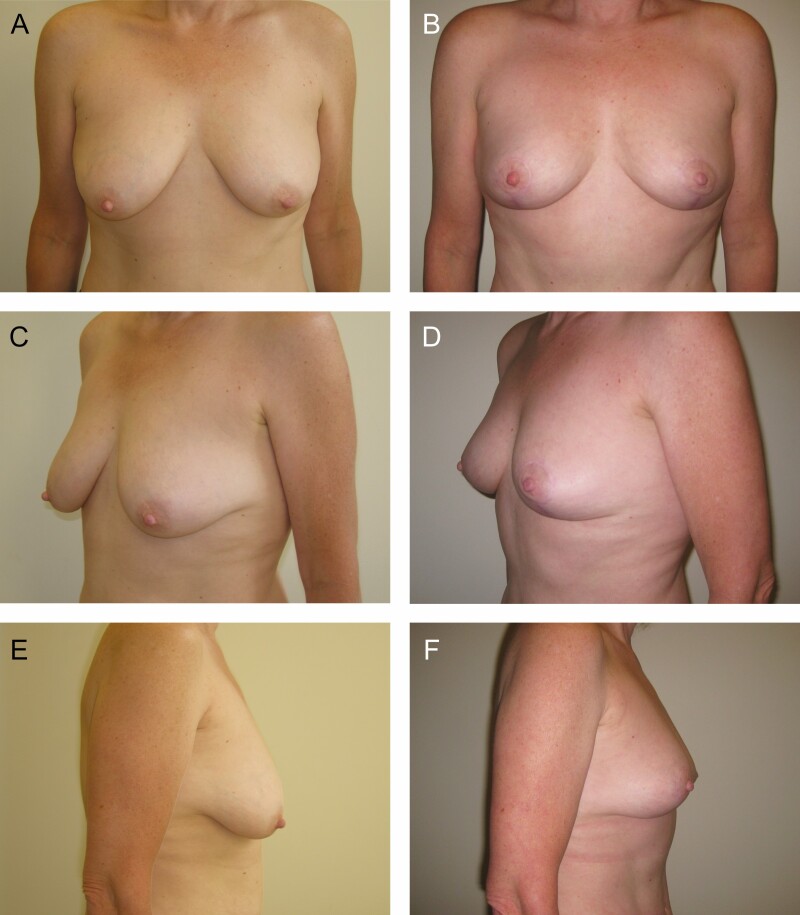
Preoperative (A, C, E) and 7-month postoperative (B, D, F) photographs of a 47-year-old female who underwent vertical scar mastopexy with a centrally based auto-augmentation flap using bilateral superior pedicles to reposition the nipple-areola complexes. She had 5 g excised from the right and 5 g excised from the left.

**Figure 7.**
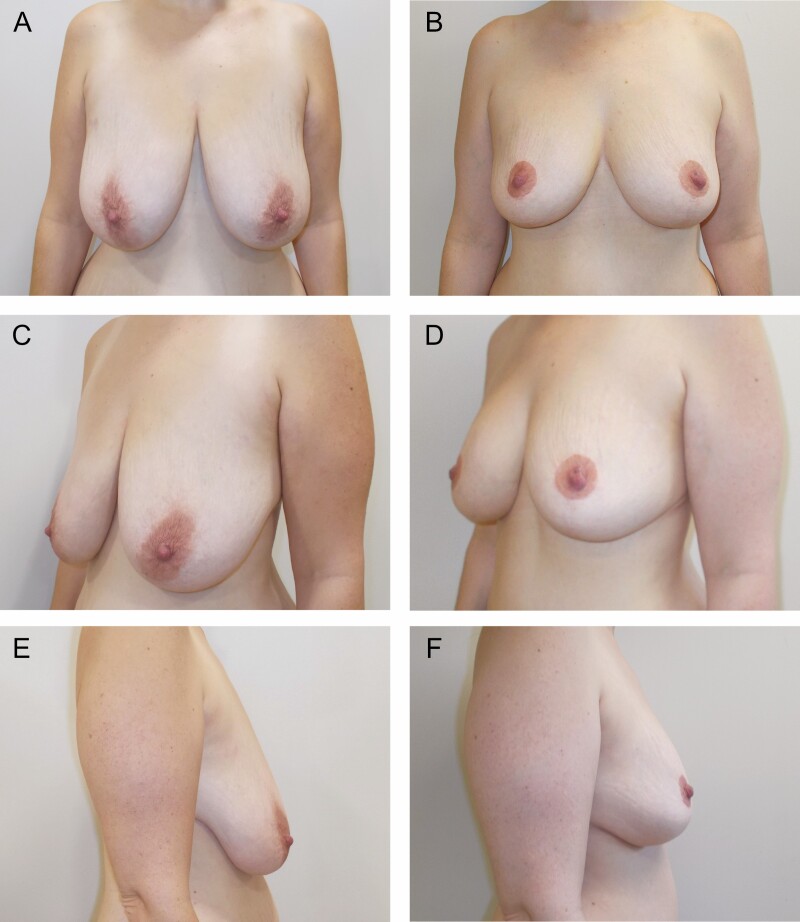
Preoperative (A, C, E) and 19-month postoperative (B, D, F) photographs of a 38-year-old female who underwent vertical scar mastopexy with a centrally based auto-augmentation flap using bilateral superior pedicles to reposition the nipple-areola complexes. She had 93 g excised from the right and 91 g excised from the left.

We performed a retrospective review of all patients who underwent vertical scar mastopexy with a centrally based auto-augmentation flap by the 3 senior surgeons (F.L., J.A., and R.E.A.). We identified 212 consecutive female patients between January 2011 and December 2021 (Figures 4-7). The mean age of the patients was 40 years (range, 17-64 years), and the mean BMI was 24.9 kg/m^2^ (range, 15.1-38.1 kg/m^2^). Twenty-two patients (10.4%) were smokers but quit 1 month before surgery. The average length of follow-up was 466 days (range, 14-3306 days).

One hundred and twenty-six patients had a mastopexy alone, whereas 86 patients (40.6%) had mastopexy combined with another procedure—abdominoplasty and liposuction being the most common. One hundred and sixty-two patients (76.4%) had a superior pedicle used bilaterally, 37 patients (17.5%) had superomedial pedicle used bilaterally, 3 patients (1.4%) superolateral pedicle used bilaterally, and 10 patients (4.7%) had a combination of pedicle types due to underlying breast asymmetry. The average weight of tissue excised per breast was 26 g (range, 0-202 g). Thirty-four patients (16.0%) had concurrent lateral chest liposuction with an average of 102 mL of liposuction per breast (range, 20-750 mL).

Eight patients (3.8%) experienced a perioperative complication (Table 2). An additional 17 patients (8.0%) underwent a subsequent procedure under local anesthesia to address aesthetic concerns, including poor scar quality, asymmetry, or an inverted nipple. There were no cases of clinically detectable fat necrosis associated with the use of the auto-augmentation flap or necrosis of the NAC.

**Table 2.** Postoperative Complications Among Patients Undergoing Vertical Scar Mastopexy With a Centrally Based Auto-Augmentation Flap

Complication	Incidence No. of patients (%)
Hematoma	3 (1.4%)
Infection	3 (1.4%)
Incidental diagnosis of breast cancer	1 (0.5%)
Pulmonary embolism	1 (0.5%)
Total	8 (3.8%)

## CONCLUSIONS

Based on our experience, vertical scar mastopexy with a centrally based auto-augmentation flap has provided good aesthetic results for patients with moderate to severe breast ptosis and adequate breast volume.
